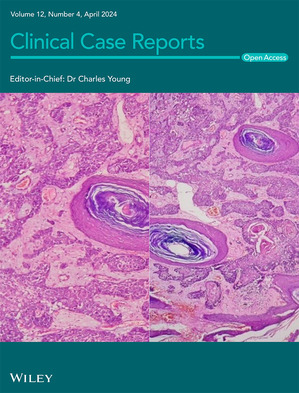# Cover Image

**DOI:** 10.1002/ccr3.8891

**Published:** 2024-05-03

**Authors:** Susmin Karki, Asmita Parajuli, Bhawesh Bhattarai, Khusbu Kumari, Kayleigh Anjali Harrylal, Pramish Bhatta, Milan K. C., Samit Sharma

## Abstract

The cover image is based on the Case Report *Neglected Fungating Giant basal cell carcinoma: A case report and literature review* by Susmin Karki et al., https://doi.org/10.1002/ccr3.8765